# Improving Sexual Safety Awareness in Kent and Medway Mental Health NHS Trust

**DOI:** 10.1192/bjo.2026.11357

**Published:** 2026-06-30

**Authors:** Indrajit Chatterjee, Sharna Bennett, Adeola Adeyemi, Elinor Bradley

**Affiliations:** 1 Kent and Medway Mental Health NHS Trust, Maidstone, United Kingdom; 2 Kent and Medway Mental Health NHS Trust, West Kent, United Kingdom; 3 Kent and Medway Mental Health NHS Trust, Dartford, United Kingdom

## Abstract

**Aims:**

Kent and Medway Mental Health NHS Trust (KMMH) is a signatory to the Sexual safety charter but despite commitments to improving workplace culture, unwanted sexual behaviour remains prevalent within different healthcare settings. National Training Survey 2025 data indicate 9% of female doctors in training experienced unwelcome sexual comments or advances causing embarrassment, distress, or offence with 6% of females in psychiatry. This quality improvement project aimed to improve awareness, confidence and engagement with sexual safety principles among resident doctors and clinical supervisors within KMMH.

**Methods:**

Two surveys were sent to trainees, locally employed doctors and consultants. The survey included questions around awareness of the Trust’s sexual safety policy, confidencearound escalating concerns, and confidence about whether concerns would be dealt with appropriately. The survey to the consultant group included similar questions around confidence in escalating resident doctor’s concerns appropriately. The surveys also included a option to provide additional comments.

The interventions included multiple sessions of Active Bystander training and Bullying, Harassment, Sexual Safety Seminar, additional workshop for lead clinical staff of the heads of school and wider deanery. A flowchart for the escalation process was also circulated.

A second survey was also sent out to the previous groups.

**Results:**

The total number of responses in the second survey were lower with fewer trainees in the second survey reporting being aware of the sexual safety policy. A small number reported attending the Bystander training and no reported attendances for the seminar. Higher numbers of consultants reported awareness (31% more compared to baseline) of the policy, with higher attendances in the interventions but less confidence (12.5% less than baseline) in the escalation process at follow-up. Trainee awareness of policy (10.5 to 0%), knowing who to escalate (12% more unconfident) and confidence in escalation process had dropped (36% more unconfident).

**Conclusion:**

This project highlights a gap between sexual safety policy and doctors’ engagement with the policy with no question to support an understanding of whether the responding trainees had seen the flow chart or attended the interventions. While consultants demonstrated higher awareness, but lower confidence in escalation processes, engagement among trainees and locally employed doctors remained limited, with lower attendance at interventions and reduced policy awareness. Development of psychologically safe environments are required with future work focused on collaborative working with keystakeholders, improved communication and protected training time to ensure sexual safety principles are embedded across all professional grades.

